# Regional myocardial strain analysis via 2D speckle tracking echocardiography: validation with sonomicrometry and correlation with regional blood flow in the presence of graded coronary stenoses and dobutamine stress

**DOI:** 10.1186/s12947-019-0183-x

**Published:** 2020-01-15

**Authors:** John C. Stendahl, Nripesh Parajuli, Allen Lu, Nabil E. Boutagy, Nicole Guerrera, Imran Alkhalil, Ben A. Lin, Lawrence H. Staib, Matthew O’Donnell, James S. Duncan, Albert J. Sinusas

**Affiliations:** 1 Section of Cardiovascular Medicine, Department of Medicine, Yale Translational Research Imaging Center, Yale University School of Medicine, P.O. Box 208017, Dana 3, New Haven, CT 06520 USA; 2 Department of Radiology and Biomedical Imaging, Yale University School of Medicine, New Haven, CT 06520 USA; 3 Department of Biomedical Engineering, Yale University School of Engineering and Applied Science, New Haven, CT 06520 USA; 4 Department of Bioengineering, University of Washington, Seattle, WA 98195-5061 USA

**Keywords:** Speckle tracking, Myocardial strain, Dobutamine stress echocardiography, Sonomicrometry, Perfusion

## Abstract

**Background:**

Quantitative regional strain analysis by speckle tracking echocardiography (STE) may be particularly useful in the assessment of myocardial ischemia and viability, although reliable measurement of regional strain remains challenging, especially in the circumferential and radial directions. We present an acute canine model that integrates a complex sonomicrometer array with microsphere blood flow measurements to evaluate regional myocardial strain and flow in the setting of graded coronary stenoses and dobutamine stress. We apply this unique model to rigorously evaluate a commercial 2D STE software package and explore fundamental regional myocardial flow-function relationships.

**Methods:**

Sonomicrometers (16 crystals) were implanted in epicardial and endocardial pairs across the anterior myocardium of anesthetized open chest dogs (*n* = 7) to form three adjacent cubes representing the ischemic, border, and remote regions, as defined by their relative locations to a hydraulic occluder on the mid-left anterior descending coronary artery (LAD). Additional cardiac (*n* = 3) and extra-cardiac (n = 3) reference crystals were placed to define the cardiac axes and aid image registration. 2D short axis echocardiograms, sonometric data, and microsphere blood flow data were acquired at baseline and in the presence of mild and moderate LAD stenoses, both before and during low-dose dobutamine stress (5 μg/kg/min). Regional end-systolic 2D STE radial and circumferential strains were calculated with commercial software (EchoInsight) and compared to those determined by sonomicrometry and to microsphere blood flow measurements. Post-systolic indices (PSIs) were also calculated for radial and circumferential strains.

**Results:**

Low-dose dobutamine augmented both strain and flow in the presence of mild and moderate stenoses. Regional 2D STE strains correlated moderately with strains assessed by sonomicrometry (*R*_*radial*_ = 0.56, *p* < 0.0001; *R*_*circ*_ = 0.55, *p* < 0.0001) and with regional flow quantities (*R*_*radial*_ = 0.61, *R*_*circ*_ = 0.63). Overall, correspondence between 2D STE and sonomicrometry was better in the circumferential direction (*Bias ± 1.96 SD*: − 1.0 ± 8.2% strain, *p* = 0.06) than the radial direction (5.7 ± 18.3%, *p* < 0.0001). Mean PSI values were greatest in low flow conditions and normalized with low-dose dobutamine.

**Conclusions:**

2D STE identifies changes in regional end-systolic circumferential and radial strain produced by mild and moderate coronary stenoses and low-dose dobutamine stress. Regional 2D STE end-systolic strain measurements correlate modestly with regional sonomicrometer strain and microsphere flow measurements.

## Introduction

The reliable assessment of myocardial function is fundamental to the diagnosis and characterization of ischemic heart disease. There is growing evidence that quantitative assessment of myocardial strain by two-dimensional (2D) speckle tracking echocardiography (STE) provides an incremental clinical benefit over conventional echocardiographic techniques for assessing systolic function, such as visual inspection and geometric calculation of left ventricular ejection fraction (LVEF) [1–3]. Global longitudinal strain has emerged as a dependable metric to support clinical decision-making, although it is limited by the fact that it is a unidirectional, generalized measure of function. The added ability to reliably measure regional strains in all cardiac strain directions is advantageous, especially in the evaluation of ischemic heart disease with regional dysfunction [4–6].

Unfortunately, regional strain measurements—especially those in the radial and circumferential directions—are hindered by poor reproducibility and inter-vendor variation [7, 8]. On a fundamental level, regional strains tend to be significantly more sensitive to noise and measurement error than global strains because they do not benefit from the favorable influences of averaging [4]. In addition, radial and circumferential 2D STE measurements in the short axis imaging plane tend to be more affected than long axis measurements by out-of-plane and rotational myocardial motion, [9] as well as intrinsic variation in the lateral and axial resolution of the ultrasound beam [10]. In all, there is a significant clinical need to evaluate and improve methods for assessing regional strains, especially those in the circumferential and radial directions.

In the current work, we present an acute open chest canine model that utilizes sonomicrometry, microsphere blood flow analysis, and invasive hemodynamic monitoring to analyze regional myocardial function and flow in the presence of graded coronary stenoses and low-dose dobutamine stress. Our unique model features an implanted 3D array of endocardial and epicardial sonomicrometer crystals that provides multidirectional strain analysis in the ischemic, border, and remote vascular territories, with direct comparison to quantitative regional blood flow data. We apply this model to address two major aims: 1) to evaluate a commercial 2D STE software platform (EchoInsight, Epsilon Imaging, Inc) in the challenging task of measuring regional radial and circumferential strains, and 2) to probe the fundamental relationship between regional myocardial blood flow and function in the presence of coronary stenoses and dobutamine stress. EchoInsight is a vendor-independent, semi-automated software platform that has demonstrated comparable performance in head-to-head comparisons with other vendor products, but has not been thoroughly evaluated in the measurement of regional radial and circumferential strains [7, 8, 11]. Our data illustrate the effects of coronary stenoses and low-dose dobutamine stress on the relationship between regional myocardial function and blood flow, and provide a rigorous evaluation of 2D STE in the assessment of regional myocardial strain. These findings have significant implications in the clinical assessment of regional myocardial ischemia and viability, particularly when considering the relative merits of flow and function measurements during low-dose dobutamine stress imaging.

## Methods

### Animal model and experimental setup

Studies were approved by and performed in accordance with federal guidelines and the standards of the Yale University Institutional Animal Care and Use Committee. Seven healthy male adult mongrel dogs were enrolled. All dogs were purchased from a commercial laboratory animal supplier (Marshall BioResources) and acclimated to their new environment for at least five days pre-procedure.

Briefly, dogs were induced with intravenous propofol (5–7 mg/kg) and endotracheally intubated. Mechanical ventilation (Venturi, Cardiopulmonary Corp.) with isoflurane (1.5–2.0%) and a mixture of nitrous oxide (45–55%) and oxygen was maintained for the duration of the experiments. Anesthesia levels were monitored and adjusted according to heart rate, blink reflex, and jaw tone. Electrocardiogram (ECG), pulse oximetry, and rectal temperature were continuously monitored. Bilateral femoral arteries and veins were cannulated for blood sampling and administration of fluids and drugs.

**Fig. 1:**
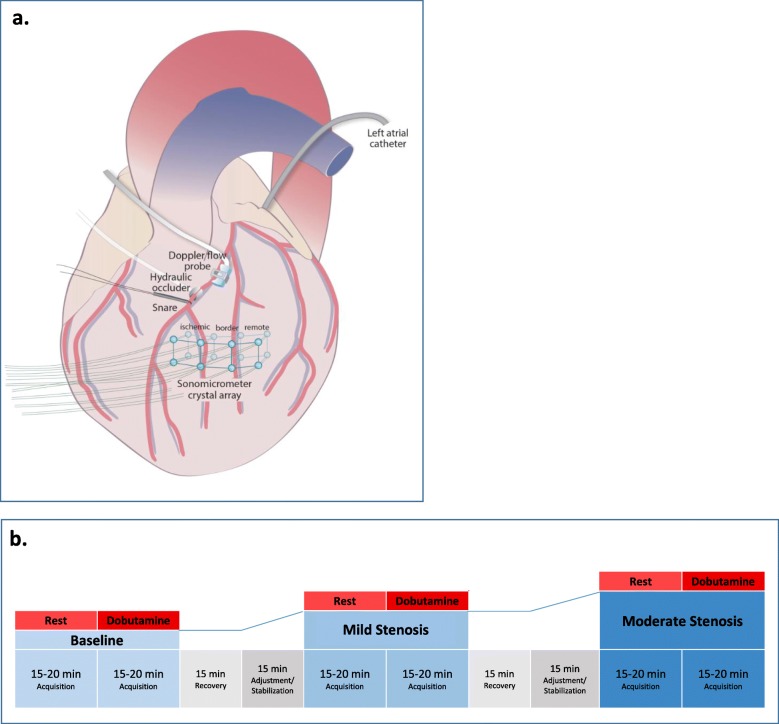
**a** Experimental open chest preparation with dissection of the LAD distal to the first diagonal and incorporation of a Doppler flow probe, hydraulic occluder, and snare. The 16-crystal sonomicrometer array contains pairs of subepicardial and subendocardial crystals which define cubic regions in the ischemic, border, and remote LAD territories. A left atrial catheter was also inserted for microsphere administration. **b** Schematic of experimental protocol. Data acquisitions at each experimental condition include invasive blood pressures, doppler LAD flow measurements, 2D echocardiography, sonimicrometry, and microsphere blood flow analysis

In each animal, an incision was made in the 5th intercostal space and the ribs were retracted to expose the heart. An incision was made in the pericardium and the pericardial edges were sutured to the chest to create a pericardial cradle. A catheter was placed in the left atrial appendage for microsphere injection. The mid-left anterior descending (LAD) coronary artery was then isolated distal to the first diagonal branch by gentle dissection. The following hardware was then attached to the mid-LAD (Fig. 1a): a) ultrasonic flow probe (Transonic, Inc.), b) snare occluder, and c) hydraulic occluder (In Vivo Metric Biomedical Products, Inc.) with micrometer for fine stenosis adjustment. A high-fidelity micromanometer catheter with dual sensors (10 cm apart; Millar, ADInstruments, Inc.) was also introduced via the right carotid artery and positioned across the aortic valve for continuous monitoring of LV and central aortic pressures. All hemodynamic data were streamed to a workstation with software that permitted continuous acquisition and off-line analysis (LabChart 8.0, AD Instruments).

Sonomicrometer crystals (Sonometrics Corp.) were then implanted in a standard configuration to assist with regional myocardial strain assessment and image registration. Cardiac reference crystals (2 mm diameter, *n* = 3) were implanted subepicardially in the apex, anterior base, and posterior base to aid in definition of the cardiac axes. Additional extracardiac reference crystals (2 mm diameter, n = 3) were attached via Parafilm (Bemis Company, Inc) to the ultrasound probe (X7–2 transducer, Koninklijke Philips) at the level of the transducer element. Myocardial crystals (1 mm diameter, *n* = 16) were then implanted in subepicardial and subendocardial pairs across the anterior wall to form three adjacent cubic elements to represent the ischemic, border, and remote regions. Placement of the myocardial crystals to define the ischemic, border, and remote regions was guided visually by the LAD anatomy and the location of the mid-LAD hydraulic occluder in relation to the diagonal branches (Fig. 1). Sutures were used to secure subepicardial crystals to the surrounding tissue. All crystals were connected via wires to a central processing box (Sonometrics Corp.).

### Data acquisition protocol

Physiologic responses to graded LAD stenoses with and without low-dose dobutamine stress were then assessed at each of the following experimental conditions: a) baseline, b) mild stenosis, c) mild stenosis with dobutamine (5 μg/kg/min), d) moderate stenosis, and e) moderate stenosis with dobutamine (5 μg/kg/min) (Fig. 1b). In each case, the flow probe/snare system was used to determine and monitor the severity of the stenosis created by the hydraulic occluder. *Mild stenosis* was defined by the absence of reduction in resting flow and a minimal hyperemic response following a complete, 10 s snare occlusion. *Moderate stenosis* was defined by an intermediate reduction in resting flow. Approximately 15 min. of serial occluder adjustments were typically required at each stenosis grade to achieve the targeted steady state flow rates. During subsequent data acquisition, occasional occluder readjustments were needed to overcome autoregulatory changes. Stenoses were not released between testing conditions. Dobutamine infusions were typically maintained for a total of 15–20 min. to allow for initial physiological stabilization and subsequent data acquisition. After discontinuation of dobutamine, a minimum washout period of 15 min. was instituted to permit return to steady state.

Open chest 2D and 3D echocardiographic images, sonomicrometer crystal displacements, and physiologic data (LV pressure, aortic pressure, ECG, Doppler LAD flow rate) were acquired at each of the above testing conditions. Echocardiographic images were acquired with a Philips iE33 Ultrasound (Koninklijke Philips) using an X7–2 transducer (B-mode, mean frequency ~ 5 MHz) at frame rates ≥60 Hz. To aid image acquisition, a flexible and transparent plastic membrane mounted on a ring stand and filled with water was placed in direct contact with the exposed heart. The transducer was then suspended in the water bath over the heart by a Buret clamp to maintain constant positioning throughout data acquisition. Sonomicrometer data were acquired with a temporal resolution of approximately 150 Hz. The intervention and acquisition sequence described above was applied in similar fashion to each animal.

### Sonomicrometer analysis and strain calculation

**Fig. 2:**
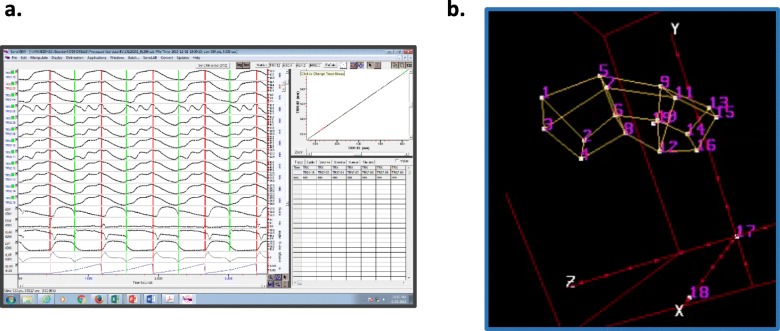
**a**) Raw sonomicrometer displacement and hemodynamic curves for a given acquisition. Each acquisition generated 462 displacement curves to account for each pair combination of the 22 crystals. **b**) Video [image] of cycle-dependent crystal positions as determined by 3D crystal coordinate solutions of the displacement curves

SonoXYZ software (Sonometrics, Inc) was used to filter and analyze raw sonomicrometer crystal displacements (Fig. 2; 462 total displacement tracings per acquisition) and generate time-dependent 3D coordinate solutions for each of the 22 crystals in the configuration (Additional file 2: Video S1). A continuum mechanics model described by Waldman [12] was adapted to 3D space and applied to the 3D coordinate solutions to calculate principle strains in the ischemic, border, and remote regions [13]. The cardiac reference crystals in the apex and base were then utilized to define cardiac axes and calculate radial and circumferential strains from the principle strains [14]. Strains were regionally averaged to provide mean values for the ischemic, border, and remote regions. End-diastole (ED) (t = 0) was defined by the upstroke of the LV pressure curve and end systole (ES) was defined by the dicrotic notch on the aortic pressure curve. End-systolic strains were calculated for each sonomicrometer strain curve.

### Semi-automated 2D echocardiographic strain analysis

**Fig. 3:**
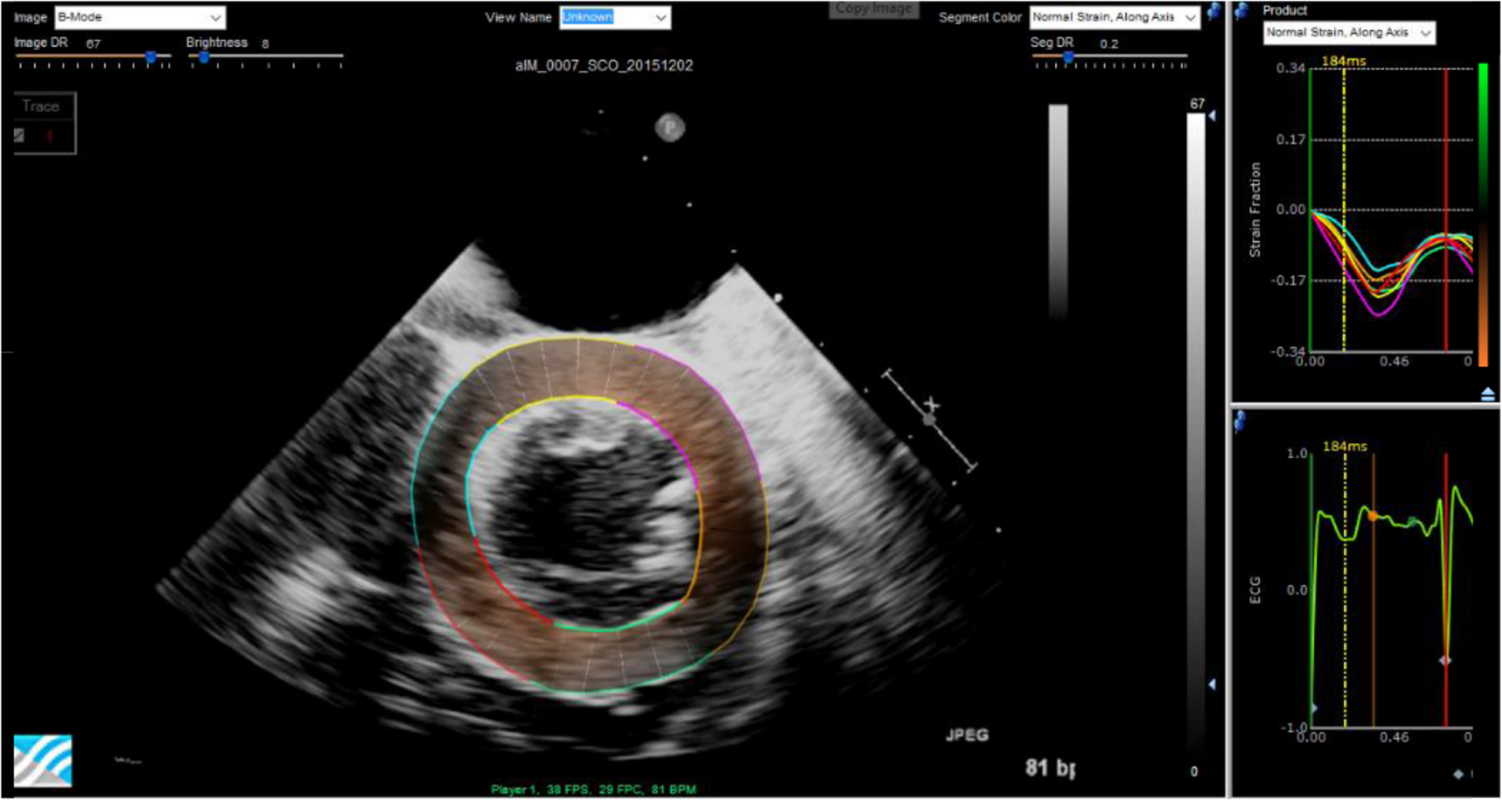
Semi-automated regional strain analysis of a short axis echocardiogram using commercial 2D STE software (EchoInsight, Epsilon Imaging), with definition of the endocardial and epicardial borders (left), and corresponding regional circumferential strain curves (upper right) and ECG (lower right)

Radial and circumferential myocardial strains were calculated over the entire cardiac cycle from mid-level short axis 2D echocardiograms with semi-automated commercial 2D STE software (EchoInsight, Research Version 2.2.51632, Epsilon, Inc.) (Fig. 3). Software-generated tracings of the endocardial and epicardial borders were manually adjusted as needed to ensure proper border definition and tracking. In accordance with American Society of Echocardiography (ASE)/European Association of Cardiovascular Imaging (EACVI)/Industry Task Force recommendations, ED was defined by the peak of the QRS complex on ECG [15]. The systolic cycle length from invasive pressure measurements was used to determine ES and aid in the calculation of end-systolic strain. Radial strain curves and both epicardial and endocardial circumferential strain curves were calculated by the software in each of 30 software defined transmural subsegments on short axis images. Tracings and strain measurements in three animals over each experimental condition (*n* = 42 circumferential, n = 42 radial) were repeated by the primary observer and then by an additional blinded observer to assess intra- and inter-observer variability (same cardiac cycles were used for repeat assessment).

**Fig. 4:**
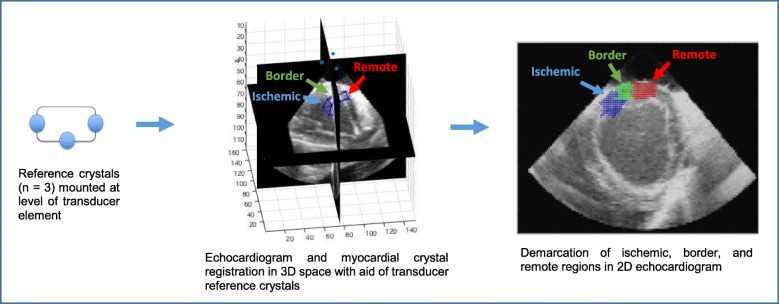
Outline of process to register the 3D ischemic, border, and remote crystal zones with 2D echocardiograms. The three transducer-mounted crystals provide a common reference in 3D coordinate space to aid registration of 3D echocardiograms and myocardial crystals (middle image). With appropriate registration, crystal zone locations can be projected onto 2D echocardiograms for regional strain analysis (right image)

To define the ischemic, border, and remote crystal zones in short axis 2D echocardiograms, 3D crystal maps determined by sonomicrometry were first registered to 3D echocardiograms (Fig. 4). Registration was aided by the echocardiographic signals of the intramyocardial crystals, as well as the three transducer-mounted crystals, which provided a common reference for the myocardial crystals and the 3D echocardiograms. With this common reference, the 3D coordinates of each myocardial crystal were computed by sonomicrometry and mapped onto the 3D echocardiogram space. Short axis planes of the registered crystal-3D echo images were then matched with corresponding 2D echocardiograms used in 2D STE analysis. End-systolic strains calculated by the 2D STE software in each of the 30 transmural subsegments were then matched to the corresponding crystal-defined ischemic, remote, and border regions. In most cases, end-systolic 2D STE strains from 2 or 3 subsegments were averaged to provide a representative value for the crystal-defined regions. In the circumferential direction, endocardial and epicardial end-systolic strains calculated by the 2D STE software were averaged to obtain strains better corresponding to the transmurally-averaged circumferential end-systolic strains determined by sonomicrometry. Post-systolic indices (PSIs) were calculated from 2D STE data by the standard formula: PSI = [(peak post-systolic strain) - (end-systolic strain)] / [peak cycle strain] [16].

### Microsphere blood flow analysis

Stable isotope labeled 10 μm polystyrene microspheres (BioPal, Inc) were also administered at each experimental condition to determine regional myocardial blood flow. Microspheres were injected via the left atrial appendage catheter and blood was sampled from two femoral arterial catheters at rates of 6 mL/min per established protocol. Following euthanasia with saturated KCl, hearts were excised and ~ 1 g tissue sections were cut from the endocardial and epicardial portions of the ischemic, border, and remote territories. Microsphere concentrations in blood and tissue samples were analyzed by neutron activation of the stable isotope labels at BioPal, Inc. and used to determine regional myocardial blood flow [mL/min/g tissue].

### Statistical analysis

Experimental quantities are expressed as means ± standard error of measurement. One-way Analysis of Variance (ANOVA) and Student’s *t*-test were used to test for statistical significance, using a threshold of *p* < 0.05 (Minitab, version 19). Linear regression and Bland-Altman analyses were employed to measure correlation and agreement between strain measurements. A total of three 2D STE strain measurements (out of 210 total) were excluded because their values exceeded three standard deviations from the linear fit of compiled 2D STE-sonomicrometer data. Intraclass correlation coefficients (ICC) were calculated for intra- and inter-observer strains. Strain-flow data were fit to logarithmic curves.

## Results

### Illustration of hemodynamic and functional responses to experimental conditions

**Fig. 5:**
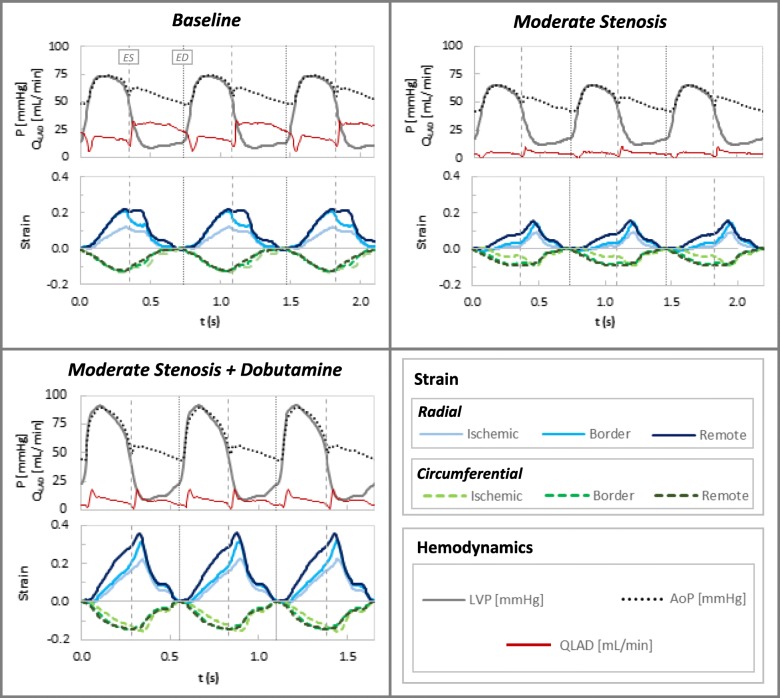
Representative hemodynamic and sonomicrometer strain curves at baseline, moderate stenosis, and moderate stenosis with dobutamine

Figure 5 presents aortic and LV pressure, Doppler LAD blood flow, and sonomicrometer-generated regional strain curves for a single representative animal at baseline, moderate stenosis, and moderate stenosis with dobutamine. Notably, the pressure, Doppler LAD flow, and strain curves each demonstrate high degrees of cycle-to-cycle reproducibility; this trend was consistent across all processed data. This figure illustrates the effect of a moderate stenosis in reducing flow and end-systolic strain and increasing post-systolic deformation, as well as the subsequent augmentation of flow and function with the addition of low-dose dobutamine.

### Hemodynamic data

Table 1 summarizes mean hemodynamic data (*n* = 7 dogs) at each experimental condition. The creation of mild stenoses in resting conditions did not substantially affect most hemodynamic variables, although there was a mild, non-statistically significant decrease in mean peak LAD blood flow rate (Q_LAD Peak_). Low-dose dobutamine stress in the presence of the mild stenoses substantially increased the magnitudes of mean aortic pressures, Q_LAD Peak_, dP/dT indices, and rate-pressure product (RPP), although there was only a modest, non-statistically significant increase in mean heart rate (HR). The creation of moderate stenoses in resting conditions substantially reduced mean Q_LAD Peak_ in comparison to baseline, while mean HR, pressures, dP/dT indices, and RPP were not significantly altered. The subsequent addition of low-dose dobutamine to the moderate stenoses significantly increased mean aortic pressures, dP/dT indices, and RPP. Mean HR and Q_LAD Peak_ also increased with the addition of dobutamine, although the augmented values were not statistically significant in comparison to corresponding baseline or moderate stenosis values. There was a non-statistically significant trend toward increasing LVEDP from initial baseline conditions to the ischemic conditions in the latter experimental stages.

**Table 1:** Hemodynamics. Compiled hemodynamic data across tested conditions (mean ± standard deviation; *HR* = heart rate, *AoP* = aortic blood pressure, *LVEDP* = left ventricular end-diastolic pressure, *Q*_*LAD Peak*_ = left anterior descending coronary artery peak Doppler blood flow rate, *dP/dT*_*max*_*, dP/dT*_*min*_ = maxima and minima of LV pressure derivative with respect to time, *RPP* = rate pressure product). Symbols denote *p* < 0.05 via one-way ANOVA († versus baseline, ‡ versus mild stenosis, § versus mild stenosis + dobutamine, ¶ versus moderate stenosis)

	HR (bpm)	AoP_Systolic_ (mmHg)	AoP_Diastolic_ (mmHg)	LVEDP (mmHg)	Q_LAD Peak_ (mL/min)	dP/dT_max_ (mmHg/s)	dP/dT_min_ (mmHg/s)	RPP (bpm*mmHg)
Baseline	93 ± 16	74.1 ± 3.9	50.9 ± 6.2	9.8 ± 3.8	42 ± 14	830 ± 150	-750 ± 130	6690 ± 1040
Mild Stenosis	92 ± 15	71.5 ± 6.1	48.6 ± 6.1	10.1 ± 2.8	25.1 ± 9.5	780 ± 190	-660 ± 160	6440 ±1130
Mild Stenosis + Dob.	105 ± 23	125 ± 20^†‡^	87 ± 17^†‡^	11.7 ± 5.4	60 ± 16^‡^	2750 ± 400^†‡^	-1860 ± 360^†‡^	13000 ± 3040^†‡^
Moderate Stenosis	95 ± 12	71.5 ± 4.4^§^	45.4 ± 1.8^§^	16.2 ± 4.8	12.6 ± 5.3^†§^	840 ± 260^§^	-610 ± 180^§^	6730 ± 1200^§^
Mod. Stenosis + Dob.	121 ± 23	109 ± 20^†‡¶^	67 ± 20^¶^	19 ± 12	31 ± 17^§^	2550 ± 570^†‡¶^	-1840 ± 680^†‡¶^	14160 ± 4690^†‡¶^

### Regional myocardial blood flow assessed with microspheres

**Fig. 6:**
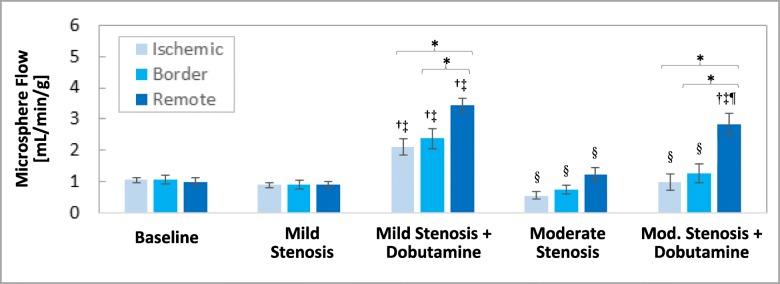
Mean regional myocardial blood flow [mL/min/g] for each experimental condition, as measured by neutron-activated microspheres. Error bars represent standard error of measurement and symbols denote *p* < 0.05 via one-way ANOVA (* versus remote (per given condition), † versus baseline, ‡ versus mild stenosis, § versus mild stenosis + dobutamine, ¶ versus moderate stenosis)

Figure 6 summarizes changes in mean regional myocardial blood flow as measured by microspheres (*n* = 7). Mean myocardial flow was similar across the ischemic, border, and remote regions at baseline, and changed very little with the creation of the mild stenoses. Mean myocardial flow increased substantially in all three regions with the addition of low-dose dobutamine to the mild stenoses, with significantly greater flow in the remote region as compared to the border and ischemic regions. The creation of moderate stenoses in resting conditions resulted in reduced mean flow in the ischemic region as compared to the remote region. The addition of low-dose dobutamine in the presence of the moderate stenoses improved flow in all three regions, although the change was only statistically significant in the remote region.

### Regional myocardial strain assessed with 2D STE and sonomicrometry

**Fig. 7:**
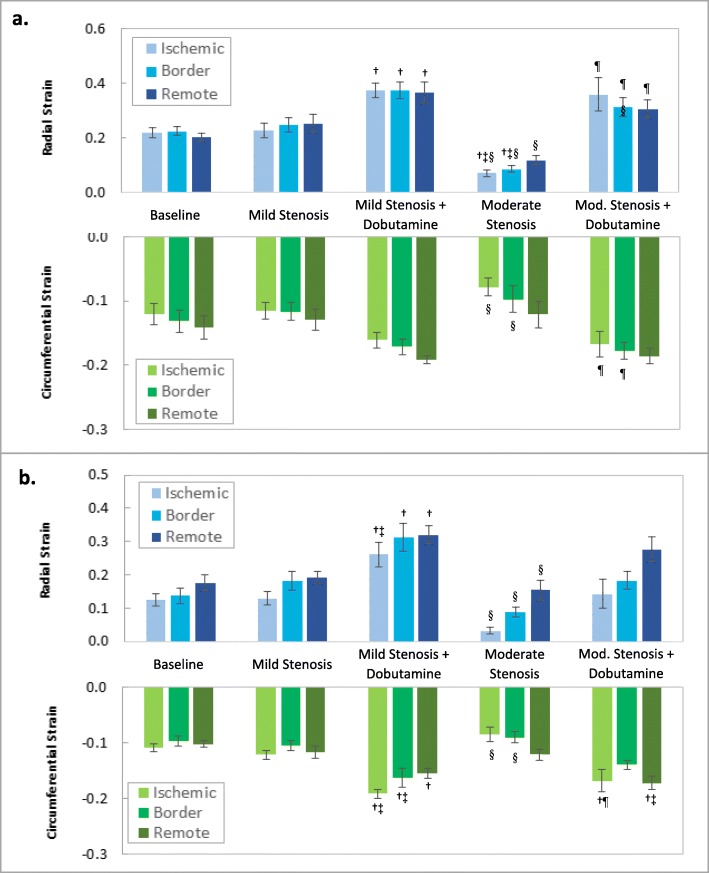
Mean regional end-systolic radial and circumferential strains (expressed as fractional values) at each experimental condition as determined by: **a** 2D STE and **b** sonomicrometry. Error bars represent standard error of measurement and symbols denote *p* < 0.05 via one-way ANOVA (* versus remote (per given condition), † versus baseline, ‡ versus mild stenosis, § versus mild stenosis + dobutamine, ¶ versus moderate stenosis)

Mean end-systolic regional radial and circumferential strains (*n* = 7; expressed as fractions) as determined by 2D STE and sonomicrometry are presented in Fig. 7a and b, respectively. Both techniques yielded similar trends in regional strain that reflect the varied physiological conditions. At baseline, both techniques demonstrated uniform mean strains across the three regions. As expected, there were minimal changes in mean strains measured by each technique after the creation of the mild stenoses, which were designed to minimize hyperemia, but not reduce resting flow or function. With the addition of low-dose dobutamine in the continued presence of the mild stenoses, both techniques demonstrated augmentation of mean radial and circumferential strains in all three regions (not all differences were statistically significant). In the presence of the moderate stenoses, both techniques demonstrated reduced mean strain magnitudes in the ischemic region when compared with baseline, although only differences in radial strains measured with 2D STE were statistically significant. Remote region strains were predictably less affected by the moderate stenoses than ischemic region strains. Finally, with the addition of low-dose dobutamine, both techniques demonstrated trends toward recovery of function, typically to mean end-systolic strain levels greater than resting baseline magnitudes (not all differences were statistically significant).

### Reproducibility of 2D STE measurements

Table 2 summarizes measures of intra- and inter-observer variability in regional end-systolic 2D STE measurements (corresponding plots in Additional file 1: Figures S1 and S2). The intraobserver analysis demonstrated good overall reproducibility in radial and circumferential strain measurements, although radial measurements were slightly less reproducible with wider limits of agreement. The interobserver analysis also demonstrated good overall reproducibility. Radial and circumferential measurements demonstrated similar levels of correlation, although radial measurements again demonstrated wider limits of agreement.

**Table 2:** Variability in 2DSTE strain measurements. Summary of intra- and inter-observer variability in 2D STE strain measurements (*R* = Pearson’s correlation coefficient, *SD* = standard deviation, *ICC* = intraclass correlation coefficient)

	Strain Measurement	*R*	Bias ± 1.96 SD	ICC
Interobserver	Circumferential	0.84	−0.013 ± 0.06	0.82
	Radial	0.85	−0.024 ± 0.15	0.84
Intraobserver	Circumferential	0.91	0.002 ± 0.04	0.87
	Radial	0.82	0.003 ± 0.22	0.75

### 2D STE-Sonomicrometry correlation and agreement

**Fig. 8:**
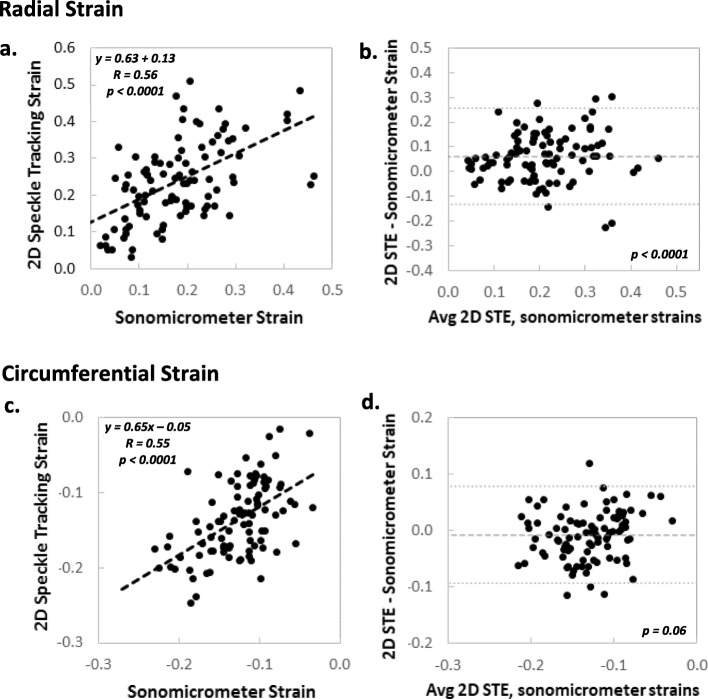
Linear regression and Bland-Altman analyses demonstrating levels of correlation and agreement between regional end-systolic strains calculated by 2D STE and sonomicrometry: **a, b** radial strains, **c, d** circumferential strains. *P-*values on Bland-Altman plots refer to differences of observed mean differences from zero and were calculated via a single sample *t*-test

Figure 8 shows linear regression and Bland-Altman analyses for all end-systolic radial and circumferential strains measured by 2D STE and sonomicrometry. There was a fair-moderate correlation for radial strains measured with the two techniques (Fig. 8a, b; *R*_*radial*_ = 0.56; *p* < 0.0001). There was a fixed bias towards greater radial strain magnitudes with 2D STE, with relatively wide limits of agreement (*Bias ± 1.96 SD*: 5.7 ± 18.3% strain, *p* < 0.0001). For circumferential strains (Fig. 8c, d), correlation between techniques was similar (*R*_*circ*_ = 0.55; *p* < 0.0001). However, there was a bias towards greater strain magnitudes with sonomicrometry, and the magnitude of the bias and the limits of agreement were less (*Bias ± 1.96 SD*: − 1.0 ± 8.2% strain, *p* = 0.06).

### Regional myocardial strain-flow relationship

**Fig. 9:**
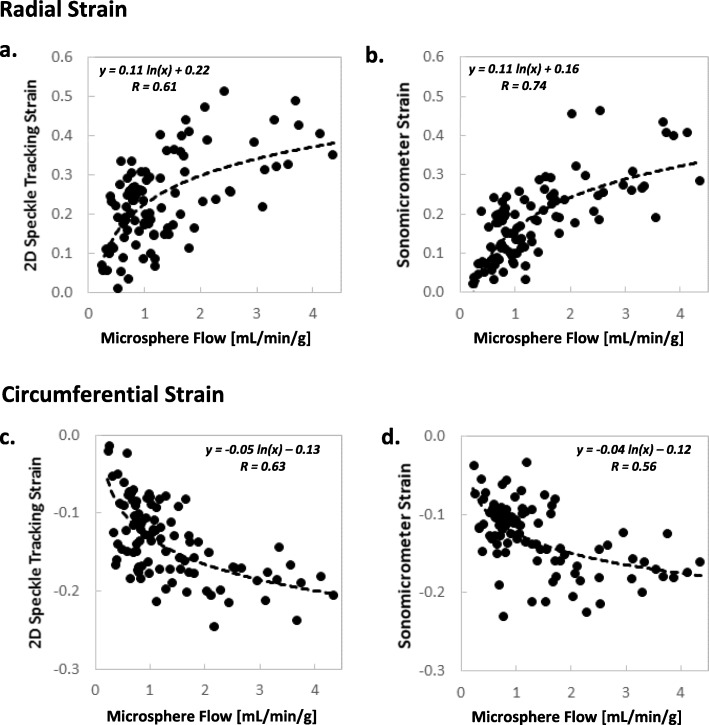
Correlation between regional end-systolic strain (2D STE and sonomicrometry) and microsphere-based blood flow: **a, b** radial strains and **c,d** circumferential strains

Figure 9 shows the relationship between end-systolic regional strains, measured by both 2D STE and sonomicrometry, and regional myocardial blood flow, measured by microsphere analysis. For both 2D STE and sonomicrometer measurements, this relationship fits logarithmic curves with correlation levels in the moderate range (*R* = 0.6–0.7).

**Fig. 10:**
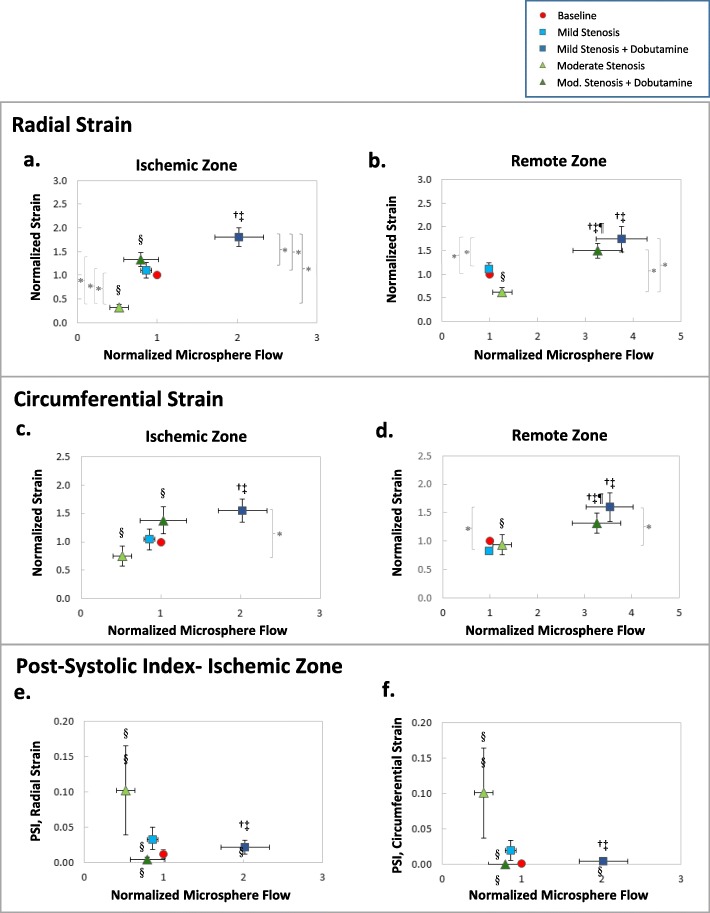
**a-d:** Comparison of mean regional end-systolic 2D STE strain and mean regional myocardial blood flow (both quantities normalized to baseline values) in the ischemic and remote territories: **a,b** radial strains, **c,d** circumferential strains. **e-f:** Comparison of mean 2D STE post-systolic indices (PSI) and mean regional myocardial blood flow in the ischemic territories: **e** radial strains, **f** circumferential strains. Error bars represent standard error of measurement and symbols denote *p* < 0.05 via one-way ANOVA (Strain, PSI: * with brackets; Blood flow: † versus baseline, ‡ versus mild stenosis, § versus mild stenosis + dobutamine, ¶ versus moderate stenosis)

Figure 10, parts a-d, summarizes the condition-dependent relationship between mean regional end-systolic 2D STE strain and mean regional myocardial blood flow (*n* = 7), with both indices normalized to their baseline values to reduce intersubject variability. This figure illustrates the degree of regional ischemic dysfunction created by graded LAD stenoses and the augmentation of flow and function produced by low-dose dobutamine. In the remote zone, magnitudes of increase from baseline due to dobutamine infusion were uniformly greater for flow measurements than strain measurements. This relationship did not hold true in the ischemic zone in the presence of moderate stenoses. Fig. 10, parts e-f, demonstrates the relationship between regional 2D STE post-systolic deformation and normalized mean regional myocardial blood flow. For both radial and circumferential 2D STE strain, PSI increased in the presence of a flow-limiting moderate stenosis, and returned to near baseline values with the addition of low-dose dobutamine.

## Discussion

Our experimental model demonstrates that 2D STE has reasonable sensitivity for identifying changes in circumferential and radial strain produced by graded coronary stenoses and low-dose dobutamine stress. Moreover, the direct fundamental relationship between regional myocardial blood flow and function was demonstrated, along with the complex physiologic effects of dobutamine. Importantly, functional changes in mean end-systolic 2D STE strain consistently reflected expected physiologic outcomes for the experimental conditions. In the presence of moderate stenoses, there was a decline in end-systolic strain with post-systolic shortening, and these changes normalized with low-dose dobutamine. Regional end-systolic 2D STE strain measurements correlated modestly with regional end-systolic strains assessed by sonomicrometry and with regional blood flow assessed by microspheres, and demonstrated moderate levels of intra- and inter-observer variability. Overall, correspondence between 2D STE and sonomicrometry was better in the circumferential direction than the radial direction. This study is the first to our knowledge to evaluate 2D strain echocardiography utilizing a complex array of sonomicrometers to provide a comprehensive analysis of both radial and circumferential strains in the ischemic, border, and remote regions, and to relate these functional measurements to regional blood flow data.

### Relation to prior studies

Prior studies utilizing sonomicrometry to evaluate 2D STE methods of regional strain assessment from short axis images in ischemic animal models demonstrate similar, moderate levels of correlation and agreement. Reant et al. utilized 3 pairs of orthogonal sonomicrometers to validate commercial speckle tracking software and reported correlation coefficients in the radial and circumferential directions that were comparable to those obtained in the current study (*R*_radial_ = 0.61, *R*_circ_ = 0.69), and greater correlation in the longitudinal direction (*R*_long_ = 0.81) [17]. The reported magnitudes of biases and limits of agreement in the radial and circumferential directions were similar to those in the current study, although both were again inferior to those reported for the longitudinal direction. By comparison, Pirat et al. utilized orthogonal pairs of mid-myocardial crystals to measure circumferential and longitudinal strains in the basal and apical anterolateral wall and reported correlations with strains from commercial feature tracking software that were slightly greater than those obtained in the current study (*R*_circ_ = 0.88, *R*_long_ = 0.83) [18]. However, the magnitudes of the biases and limits of agreement in the circumferential direction were greater than those reported here, with substantially greater bias for large magnitude strains. Similarly, longitudinal strain biases and limits of agreement in this prior study were substantially less than those for the circumferential direction. In another study, Korenic et al. utilized two separate sonomicrometer triplets to measure peak systolic circumferential and longitudinal strains in the anteroseptal and midposterior regions and reported similar levels of correlation at baseline (*R*_circ_ = 0.68, 0.65; *R*_long_ = 0.71, 0.73). However, in the setting of ischemia, correlation in the circumferential direction was substantially reduced (*R*_circ_ = 0.19) [19].

Collectively, the results of the current study and these prior experiments suggest that regional end-systolic circumferential 2D STE strain measurements correlate and agree with reference sonomicrometer values at levels that are slightly more favorable than regional radial measurements. Longitudinal 2D STE strain analysis was not performed in the current study because the open chest preparation does not permit standard apical acquisitions to assess longitudinal strain. However, the observed correlations between 2D STE and sonomicrometer strains in the radial and circumferential directions were generally less than those reported for longitudinal strains in the other studies [17–19]. As noted above, greater out-of-plane motion in the short axis image plane may contribute to these differences between short and long axis measurements [9]. Of course, interstudy data comparison is limited by many factors, including differences in speckle-tracking methods and sonomicrometer crystal configurations.

In the current study, the 3D 16-crystal intramyocardial sonomicromter array and associated cardiac and extracardiac reference crystals improves registration with echocardiographic images and better estimates strains in standard cardiac axes than the limited crystal groupings employed in previous studies. The sonomicrometer array uniquely provides a comparative evaluation of regional strains in multiple directions and vascular territories. Our model further relates these strains to regional microsphere-based flows, providing physiological information that is not provided by prior imaging studies. We believe that accurate delineation of the ischemic, border, and remote zones by the sonomicrometer crystals in each experiment is confirmed by the corresponding microsphere blood flow data.

### Relationship between regional myocardial function and blood flow

A key outcome of our study is a direct illustration of the complex, non-linear relationship between regional myocardial function and blood flow, and demonstration of how this relationship is affected by dobutamine stress. The observed logarithmic strain-flow relationship is consistent with prior studies demonstrating non-linearity in this relationship over the normal physiological range [20]. This likely reflects intrinsic physiological and mechanical properties of myocardium, including the potentially prominent influences of loading conditions and passive tissue forces, especially in ischemic tissue with reduced contractility [21]. In addition, the baseline strain-flow relationship in this study is altered by dobutamine, which differentially affects inotropy and vasodilation through various mechanisms [22].

The moderate stenoses produced significant regional dysfunction, but typically did not lead to substantial reductions in systolic aortic pressure. This indicates that the ischemic burden from the moderate stenoses was in the intermediate range and reflects the location and degree of the stenoses, as well as the relatively high level of collateral circulation in canine hearts. By comparison, low-dose dobutamine (5 μg/kg/min) improved myocardial blood flow and function in all regions in the presence of both mild and moderate stenoses. While dobutamine at this dose clearly increased demand—as evidenced by RPP augmentation—further ischemic dysfunction was not created. Increases in demand from the inotropic stimulation were at least partially offset by concomitant increases in myocardial blood flow due to increased driving pressure and the direct and indirect vascular effects of dobutamine [22]. This pharmacological augmentation of flow and function in ischemic myocardium is fundamental to clinical viability assessment with low-dose dobutamine stress echocardiography (DSE). Accurate detection and localization of stress-induced changes in regional myocardial function with DSE is critical, as the information helps to determine prognosis and guide procedures such as coronary revascularization [23].

The non-linearity of the flow-function relationship has significant implications when considering the relative merits of flow and function measurements in clinical assessments of ischemia and viability. Our data illustrate that quantitative regional microsphere blood flow was more sensitive than strain to detect differences between ischemic and remote regions in the presence of graded ischemia and low-dose dobutamine stress. While microsphere-based flow measurement is not a clinical technique, this finding aligns with the general assertion that clinical measurements of regional blood flow tend to be slightly more sensitive than measurements of regional strain for the detection of obstructive coronary stenoses [24, 25]. This likely reflects intrinsic myocardial physiology and the well-established ischemic cascade, as well as differences in the sensitivity of clinical techniques for measuring regional myocardial strain and flow. Despite this observation, quantitative regional strain analysis by 2D STE offers several advantages over radiotracer-based perfusion imaging, including its lesser cost, greater availability, and lack of ionizing radiation, as well as potentially greater specificity for the detection of obstructive coronary artery disease [24, 25]. The addition of reliable, quantitative regional and multidirectional strain analysis to traditional stress echocardiography may not only improve diagnostic accuracy, but also potentially enhance quantification of disease severity and risk stratification.

### Challenges associated with assessment regional radial and circumferential strain

While our results illustrate the potential clinical utility of 2D STE to measure regional strain in the circumferential and radial directions, they also highlight some of the fundamental challenges associated with these measurements. As noted above, decreased speckle pattern stability in short axis images relative to those in the long axis is likely a key factor affecting accuracy in 2D STE circumferential and radial strain measurements [26]. Variability in 3D STE radial and circumferential strain measurements has been shown to be comparatively less, presumably due to better tracking of out-of-plane motion [26]. However, 3D echocardiography is less available in the clinical setting and current technologies typically provide inferior temporal resolution as compared to 2D acquisitions. Interestingly, a recent sonometric validation study of 3D STE demonstrated good correlation and agreement for longitudinal and circumferential strains, but poor correlation and agreement for radial strains [27].

2D STE can be particularly sensitive to image quality and the temporal and spatial resolution of the acquisitions. In this study, 2D echocardiograms were acquired on open chest hearts at frame rates ≥60 Hz and spatial resolutions on the order of 2–5 mm. Our research ultrasound system has been modified to allow for 2D acquisitions at frame rates exceeding 60 Hz, the traditional limit on clinical systems due to probe heating. Although vendor-independent software platforms such as EchoInsight can analyze data from multiple acquisition sources and are thus advantageous for standardization of clinical 2D STE measurements, [28] it is critical that analyses are not performed on down-sampled server-based DICOM images. Under-sampling due to inadequate frame rates can significantly reduce frame-to-frame speckle continuity [29] and typically leads to underestimation of strains, especially at fast heart rates [30]. The optimal frame rate for speckle tracking analysis remains a trade-off, however, as greater frame rates are typically associated with lower spatial resolution and signal-to-noise ratios [30, 31]. Overall, image quality remains one of the most important determinants of 2D STE tracking proficiency and is a potentially significant source of interoperator variability. In addition to efforts to improve image quality and temporal resolution through better instrumentation and data processing, numerous efforts are also underway to improve tracking methods. Recent advancements include algorithms that integrate shape and speckle tracking, utilize radiofrequency data, and employ machine learning [13, 32–35].

Cardiac cycle definition is another factor that can limit the accuracy and reproducibility of end-systolic strain measurements from mid-level short axis echocardiograms, as these images do not show opening and closing of the aortic and mitral valves. This is less relevant in the current study because ES was defined by invasive pressure measurements, but is highly relevant in clinical settings where such hemodynamic data are typically not available. While indirect surrogates of ES such as maximum global strain and t-wave onset have been shown to be reasonably reliable when applied in non-diseased hearts, they are known to be potentially significant sources of error in the presence of myocardial dysfunction and altered QRS morphology [36]. Most often, end-systolic strains defined by indirect approximations of ES tend to be overestimated due to the presence of post-systolic deformation related to delayed myocyte contraction and/or passive mechanisms [21]. This potential for timing error within the cardiac cycle is known to be even greater in regional strain measurements than global measurements [36]. Ultimately, the only definitive, non-invasive technique to define cycle timing in short axis images is to acquire concurrent parasternal long axis or Doppler flow images that objectively demonstrate aortic and mitral valve positions. While peak strain can be reported instead of end-systolic strain, it is typically less sensitive for the detection of ischemia due to post-systolic deformation. We observed post-systolic shortening in the presence of moderate stenoses that normalized with the addition of low-dose dobutamine.

### Experimental limitations

Sonomicrometry is regarded as a gold standard for the determination of strain because it provides accurate deformational data with high levels of spatiotemporal resolution [10, 18, 37]. However, there is potential for error related to misalignment between the crystals and ultrasound beam and in the definition of cardiac axes. In this experiment, a system of cardiac and extracardiac reference crystals was utilized to assist with alignment and minimize these potential sources of error. In addition, the subendocardial and subepicardial crystal positioning in sonomicrometry inevitably leads to incomplete sampling of tissue closest to the endocardial and epicardial surfaces [17, 18]. Crystal positioning can potentially influence measurements given the substantial anisotropy of myocardial strain and the prominent gradient of increasing deformation from the epicardial to the endocardial surface [20]. In comparison to the relatively small numbers of crystals used in other experiments, [17, 18] the large, 3D crystal array in this experiment may help to minimize these sources of error by utilizing multiple crystal inputs to determine individual regional strain values.

Additionally, the invasive nature of this experimental model and its potential effects on myocardial function are worth noting. In general, the magnitudes of baseline radial and circumferential strains measured by both sonomicrometry and 2D STE in this study are less than those reported in clinical studies [26, 28] and other open chest canine studies [20, 38]. This finding is not entirely surprising given that a certain degree of functional impairment is expected due to the combined effects of anesthesia and myocardial instrumentation. In addition, it is likely that mechanics are altered to a small degree by the open chest preparation, the water bath suspended over the heart, and mechanical ventilation. As such, this experimental model is most appropriate for strain measurement validation and physiologic investigation of regional myocardial flow and function, rather than measurement of absolute, clinically comparable strain values.

### Clinical implications and future directions

Our findings contribute to the foundation of knowledge in the developing clinical application of quantitative regional and multidirectional strain analysis. The demonstration of a non-linear relationship between regional myocardial function and blood flow has significant implications when considering the relative virtues of strain and perfusion imaging in clinical assessments of ischemia and viability. In addition, our study provides a rigorous, in-depth evaluation of a clinical 2D STE software package. Our results indicate that 2D STE requires additional refinement before becoming a reliable quantitative clinical technique for measuring regional circumferential and radial strains. Reassessment of FDA-approved clinical software for strain analysis is necessary to determine both clinical merits and limitations. Ultimately, continued collaboration between leaders in ASE, EACVI, and industry is fundamental for coordinating efforts to improve methodology, instrumentation, image processing, and tracking algorithms in order to advance regional 2D and 3D STE measurements towards greater clinical utility [15].

## Conclusions

The unique experimental model that we present here illustrates the fundamental relationship between regional myocardial blood flow and function and demonstrates that 2D STE identifies regional changes in circumferential and radial strain produced by graded coronary stenoses and low-dose dobutamine stress. Additionally, our model shows that regional circumferential and radial strains measured by 2D STE correlate and agree modestly with the gold standard of sonomicrometry and have reasonable levels of inter- and intra-observer reproducibility. While these findings are encouraging, there is a clear clinical need to improve regional radial and circumferential strain measurement techniques.

## Supplementary information

**Additional file 1: Figure S1.** Linear regression and Bland-Altman analyses demonstrating intraobserver correlation and agreement for 2D STE strains: **a, b** radial strains, **c, d** circumferential strains (note: ‘Obs 1a’ and ‘Obs 1b’ refer to strain measurements performed in duplicate by the same observer). *p-*values on Bland-Altman plots refer to deviations of observed mean strain differences from zero and were calculated via a single sample *t*-test. **Figure S2:** Linear regression and Bland-Altman analyses demonstrating interobserver correlation and agreement for 2D STE strains: **a, b** radial strains, **c, d** circumferential strains. (note: ‘Obs 1’ and ‘Obs 2’ refer to strain measurements performed in duplicate by two separate observers). *p-*values on Bland-Altman plots refer to deviations of observed mean strain differences from zero and were calculated via a single sample *t*-test.

**Additional file 2:** **Video S1.** Cycle-dependent crystal positions as determinded by 3D crystal coordinate solutions of the displacement curves.

## Data Availability

The datasets used and/or analyzed during the current study are available from the corresponding author on reasonable request.

## Abbreviations

2D: Two-dimensional
3D: Three-dimensional
ASE: American Society of Echocardiography
DSE: Dobutamine stress echocardiography
EACVI: European Association of Cardiovascular Imaging
ED: End diastole
ES: End systole
HR: Heart rate
ICC: Intraclass correlation coefficient
LAD: Left anterior descending coronary artery
LVEDP: Left ventricular end diastolic pressure
LVEF: Left ventricular ejection fraction
RPP: Rate-pressure product
STE: Speckle tracking echocardiography
