# Supplementary material for: Regional myocardial strain analysis via 2D speckle tracking echocardiography: validation with sonomicrometry and correlation with regional blood flow in the presence of graded coronary stenoses and dobutamine stress
Source: Cardiovasc Ultrasound. 2020 Jan 15;18:2. doi: 10.1186/s12947-019-0183-x (PMC6964036; doi:10.1186/s12947-019-0183-x)

**Supplemental Material**

**Supplemental Figure Legends**

**Supplemental Figure 1:** Linear regression and Bland-Altman analyses demonstrating intraobserver correlation and agreement for 2D STE strains: **(a, b)** radial strains, (**b, c)** circumferential strains (note: ‘Obs 1a’ and ‘Obs 1b’ refer to strain measurements performed in duplicate by the same observer). *p-*values on Bland-Altman plots refer to deviations of observed mean strain differences from zero and were calculated via a single sample *t*-test.

**Supplemental Figure 2:** Linear regression and Bland-Altman analyses demonstrating interobserver correlation and agreement for 2D STE strains: **(a, b)** radial strains, (**b, c)** circumferential strains. (note: ‘Obs 1’ and ‘Obs 2’ refer to strain measurements performed in duplicate by two separate observers). *p-*values on Bland-Altman plots refer to deviations of observed mean strain differences from zero and were calculated via a single sample *t*-test.

**Supplemental Figure 1**

**Circumferential Strain**

**Radial Strain**


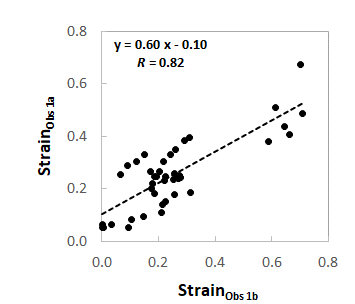

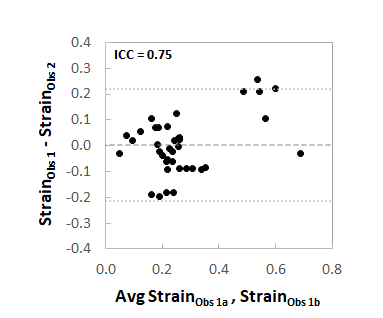


***p* < 0.0001**

**Strain_Obs1a_ - Strain_Obs1b_**

**a.**

**b.**

***p* = 0.11**

**c.**

**d.**


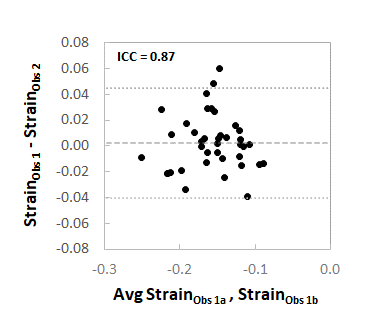

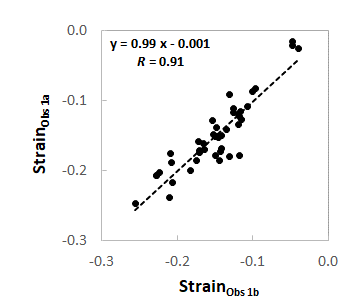


***p* = 0.51**


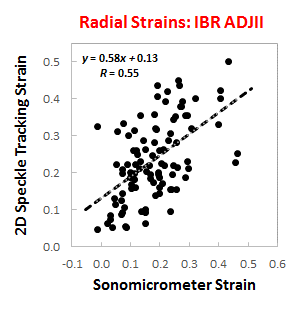

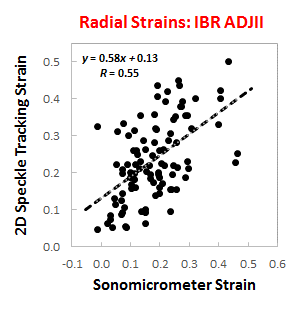


***p* < 0.0001**

**Strain_Obs1a_ - Strain_Obs1b_**

**Supplemental Figure 2**

**Circumferential Strain**

**Radial Strain**


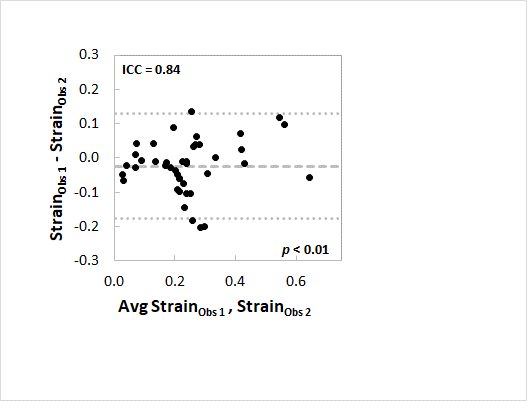

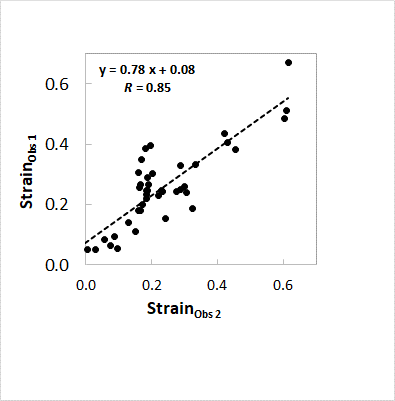


***p* < 0.0001**

**a.**

**b.**


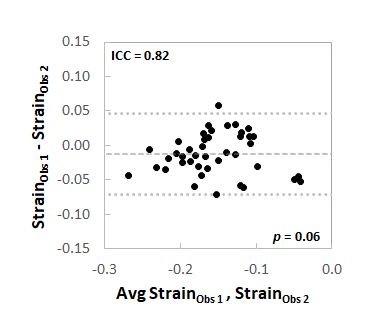


***p* < 0.0001**

**c.**

**d.**


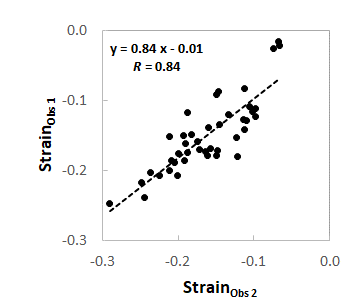


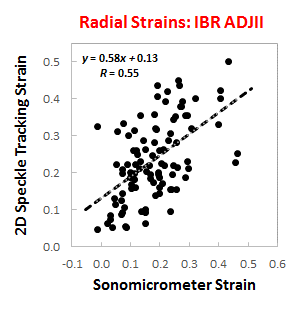

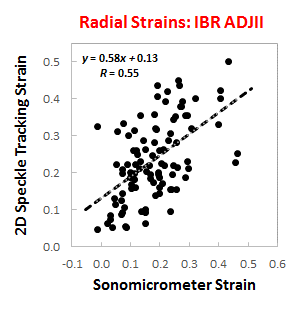

Supplement: Supplementary file 1 — Additional file 1: Figure S1. Linear regression and Bland-Altman analyses demonstrating intraobserver correlation and agreement for 2D STE strains: a, b radial strains, c, d circumferential strains (note: ‘Obs 1a’ and ‘Obs 1b’ refer to strain measurements performed in duplicate by the same observer). p-values on Bland-Altman plots refer to deviations of observed mean strain differences from zero and were calculated via a single sample t-test. Figure S2: Linear regression and Bland-Altman analyses demonstrating interobserver correlation and agreement for 2D STE strains: a, b radial strains, c, d circumferential strains. (note: ‘Obs 1’ and ‘Obs 2’ refer to strain measurements performed in duplicate by two separate observers). p-values on Bland-Altman plots refer to deviations of observed mean strain differences from zero and were calculated via a single sample t-test. [file 12947_2019_183_MOESM1_ESM.docx]
